# Fabrication of self-assembled Au droplets by the systematic variation of the deposition amount on various type-B GaAs surfaces

**DOI:** 10.1186/1556-276X-9-436

**Published:** 2014-08-27

**Authors:** Mao Sui, Ming-Yu Li, Eun-Soo Kim, Jihoon Lee

**Affiliations:** 1 College of Electronics and Information, Kwangwoon University, Nowon-gu Seoul 139-701, South Korea; 2 Institute of Nanoscale Science and Engineering, University of Arkansas, Fayetteville, AR 72701, USA

**Keywords:** Self-assembled Au droplets, Deposition amount, GaAs type-B, Nucleation, Diffusion

## Abstract

The fabrication of self-assembled Au droplets is successfully demonstrated on various GaAs (*n*11)B, where *n* is 2, 4, 5, 7, 8, and 9, by the systematic variation of the Au deposition amount (DA) from 2 to 12 nm with subsequent annealing at 550°C. Under an identical growth condition, the self-assembled Au droplets of mini to supersizes are successfully synthesized via the Volmer-Weber growth mode. Depending on the DA, an apparent evolution is clearly observed in terms of the average height (AH), lateral diameter (LD), and average density (AD). For example, compared with the mini Au droplets with a DA of 2 nm, AH of 22.5 nm, and LD of 86.5 nm, the super Au droplets with 12-nm DA show significantly increased AH of 316% and LD of 320%, reaching an AH of 71.1 nm and LD of 276.8 nm on GaAs (211)B. In addition, accompanied with the dimensional expansion, the AD of Au droplets drastically swings on 2 orders of magnitudes from 3.2 × 10^10^ to 4.2 × 10^8^ cm^-2^. The results are systematically analyzed with respect to the atomic force microscopy (AFM) and scanning electron microscopy (SEM) images, energy-dispersive X-ray spectrometry (EDS) spectra, cross-sectional line profiles, Fourier filter transform (FFT) power spectra, and root-mean-square (RMS) roughness as well as the droplet dimension and density summary, respectively.

## Background

Interests on semiconductor nanowires (NWs) are derived from their unique physical properties compared with the bulk materials such as the quantum confinement and increased cross sections [1,2] as well as their potentials to be adapted in numerous electronic, optoelectronic, and nanomechanic applications [3-5]. For instance, a single GaAs NW photovoltaic device has demonstrated 40% conversion efficiency over the 'Shockley-Queisser limit’ [5]. The fabrication of NWs is usually achieved via the metallic droplet-assisted vapor-liquid-solid (VLS) mechanism [6-8]. In the VLS, crystallization can occur at the liquid-solid interface due to the higher sticking coefficient and the Au droplets as a common catalyst exert an excellent capability of transferring the vapor phase precursors through the supersaturation regardless of the materials and substrates utilized. Naturally, NWs can only be grown at droplet sites, and thus, the fabrication of NWs including the size, density, and shape strongly depends on the fabrication of Au droplets. For example, ZnO NWs showed a larger diameter as well as lower density with the increased size of droplets [9]. To date, various NWs such as Si, Ge, ZnO, GaN, GaAs, InP, and InAs have been fabricated by the Au droplet-assisted VLS approach [9-16]. In the meantime, due to their unique properties and applications, such as localized surface plasmonic resonance, catalysis, quantum size effect, and bio-sensing, Au droplets have drawn a lot of research attention and have been demonstrated on diverse surfaces including Si, sapphire, SiO_2_, GaN SiC, and polymeric substrates [17-25]. As a common semiconductor with a direct band gap, GaAs is widely used in light-absorbing and light-emitting devices, and also various GaAs surfaces of different indices are often used in controlled fabrication of nanostructures. For example, the cross-sectional shape of NWs can be determined by substrate indices such as a triangular shape on GaAs (111)A, trapezoid shape on GaAs (110), and hexagonal shape on GaAs (111)B [26-28]. In addition, the resulting NWs on GaAs (111)B often showed stacking faults (SFs), and SF-free NWs can be successfully fabricated on GaAs (311)B and others [29-31]. This naturally puts the investigation on the Au droplets synthesized on a diverse GaAs index, which is an essential research topic in the fabrication of desired NWs. However, to date, systematic studies on Au droplets on type-B GaAs are still deficient.

**Figure 1:**
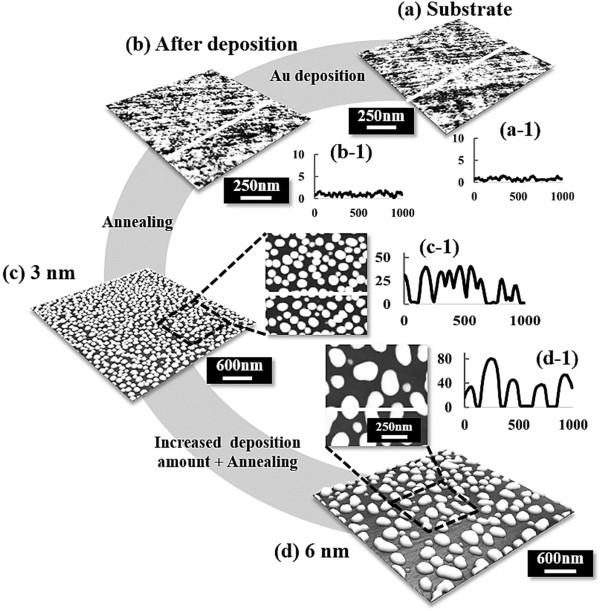
**Illustration of self-assembled Au droplet evolution on GaAs (211)B as a function of deposition amount (DA). (a)** Bare GaAs surface. **(b)** After 3-nm Au deposition. **(c)** Au droplets with 3-nm DA. **(d)** Au droplets with 6-nm DA. **(a-1)** - **(d-1)** Line profiles of the cross sections marked with the white lines on AFM top views.

In this paper, we thus demonstrate the fabrication of self-assembled Au droplets on various GaAs (*n*11)B, where *n* is 2, 4, 5, 7, 8, and 9 via the systematic variation of the Au deposition amount (DA). As an example, the simplified fabrication process of the self-assembled Au droplets on GaAs (211)B via the Volmer-Weber growth mode [32-34] is illustrated in Figure 1. Staring from the bare GaAs (211)B in Figure 1a, the surface still showed a quite smooth surface topography even after the 6-nm Au deposition as shown in Figure 1b,b-1. After a systematic annealing process, the resulting Au droplets are shown with the 3-nm deposition in Figure 1c and 6-nm DA in Figure 1d. Under an identical growth condition, the self-assembled Au droplets show drastically different sizes and densities, and as a function of the DA, a gradual dimensional expansion including the average height and the average diameter was clearly observed while the average density swings over 2 orders of magnitude. On the various substrates utilized, a similar trend of the evolution process was clearly observed while showing minor index dependency.

## Methods

In this work, the fabrication of the self-assembled Au droplets was investigated on various GaAs type-B (*n*11) substrates, where *n* is 9, 8, 7, 5, 4, and 2 in a pulsed laser deposition (PLD) system. The GaAs wafers utilized in this work were semi-insulating or undoped with an off-axis of ±0.1° from the Wafer Technology Ltd. (Milton Keynes, UK). To start with, a batch of samples including the various type-B GaAs substrates was indium soldered on an Inconel sample holder side by side to maintain the uniformity among the samples and then was treated with a 30-min degas process at 350°C under 1 × 10^-4^ Torr to remove the contaminants. Subsequently, Au deposition was equally performed on the various type-B GaAs substrates at a growth rate of 0.05 nm/s with an ionization current of 3 mA under 1 × 10^-1^ Torr in a plasma ion-coater chamber. Au deposition of 2, 3, 4, 6, 9, and 12 nm was systematically performed, and regardless of the deposition amount, the surface showed a quite smooth morphology as shown in Figure 1b,b-1. As an example, Table 1 shows the root-mean-square (RMS) roughness (*R*_q_) of the various GaAs surfaces after the 3-nm Au deposition as compared to the Figure 1b. Next, annealing process was implemented by a programmed recipe, and the substrate temperature (*T*_sub_) was gradually increased to 550°C from the ambient temperature (approximately 25°C) at a fixed rate of 1.83°C/s under a chamber pressure of 1 × 10^-4^ Torr. After reaching the target *T*_sub_ (550°C) [35], the samples were dwelt for 150 s to ensure the maturation of the droplets. Immediately after the dwell process, the samples were quenched down to the ambient temperature to minimize the ripening effect [36,37]. An atomic force microscope (AFM) under atmospheric pressure was employed to characterize the surface morphology with non-contact tapping mode. The tips used in this work were NSC16/AIBS (μmasch, Lady's Island, SC, USA) with a curvature radius less than 10 nm. The spring constant was approximately 40 N/m, and the resonation frequency was approximately 170 kHz. A scanning electron microscope (SEM) under vacuum was utilized for the characterizations of the resulting samples, and energy-dispersive X-ray spectrometry (EDS) was utilized (Thermo Fisher Noran System 7, Thermo Fisher Scientific, Waltham, MA, USA) for the elemental analysis.

**Table 1:** **Root-mean-square (RMS) roughness (*R***_**q**_**) of various GaAs surfaces after 3-nm Au deposition**

**Surface**	**(211)B**	**(411)B**	**(511)B**	**(711)B**	**(811)B**	**(911)B**
*R*_q_ [nm]	0.361	0.264	0.232	0.351	0.347	0.269

## Results and discussion

**Figure 2:**
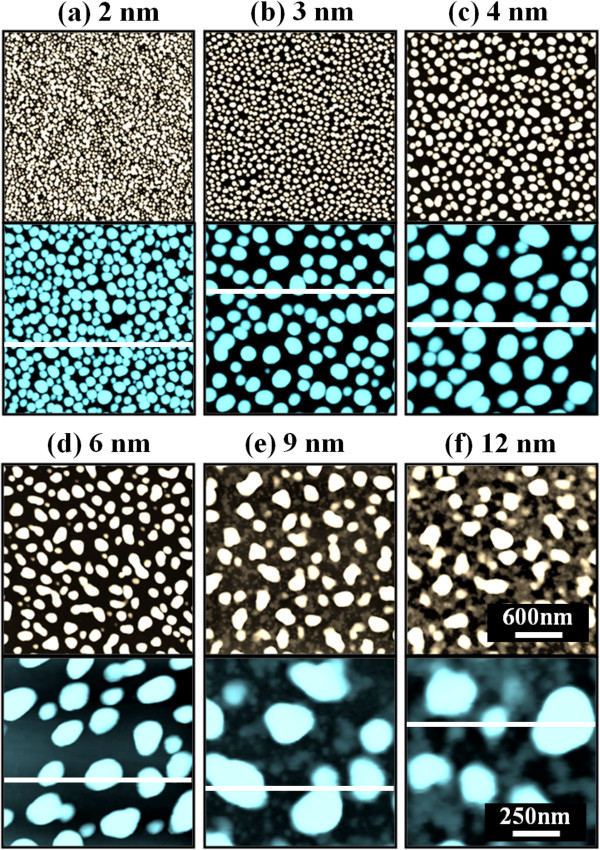
**Au droplet evolution on GaAs (211)B induced by the systematic variation of the Au DA. (a)** 2 nm, **(b)** 3 nm, **(c)** 4 nm, **(d)** 6 nm, **(e)** 9 nm, and **(f)** 12 nm. Au droplets are presented with AFM top views of 3 × 3 μm^2^ and 1 × 1 μm^2^.

**Figure 3:**
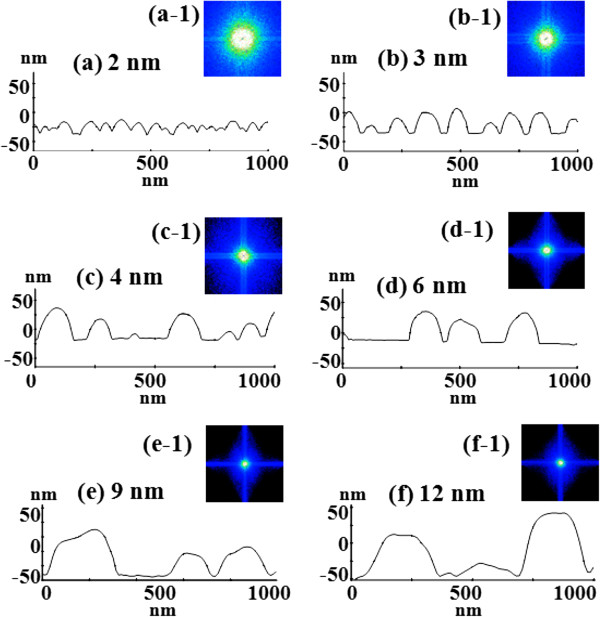
**Line profiles and corresponding FFT power spectra. (a-f)** Line profiles of the cross sections indicated with the white lines in Figure 2a,b,c,d,e,f of 1 × 1 μm^2^ AFM top views. **(a-1)** - **(f-1)** The corresponding Fourier filter transform power spectra.

**Figure 4:**
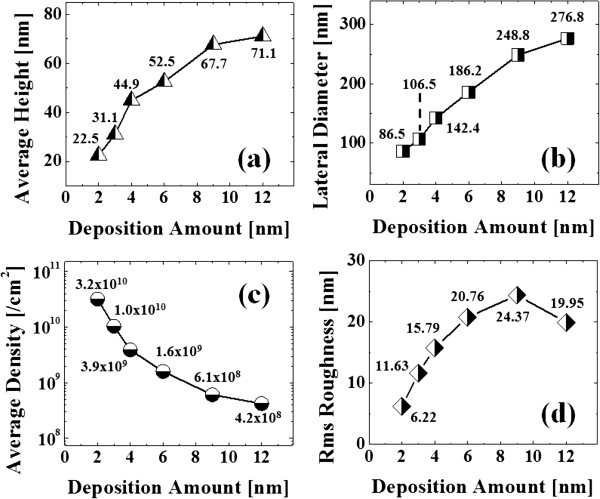
**Summary plots of self-assembled Au droplets on GaAs (211)B as a function of DA. (a)** Average height (AH), **(b)** average lateral diameter (LD), **(c)** average density (AD), and **(d)** root-mean-square (RMS) roughness (*R*_q_).

**Figure 5:**
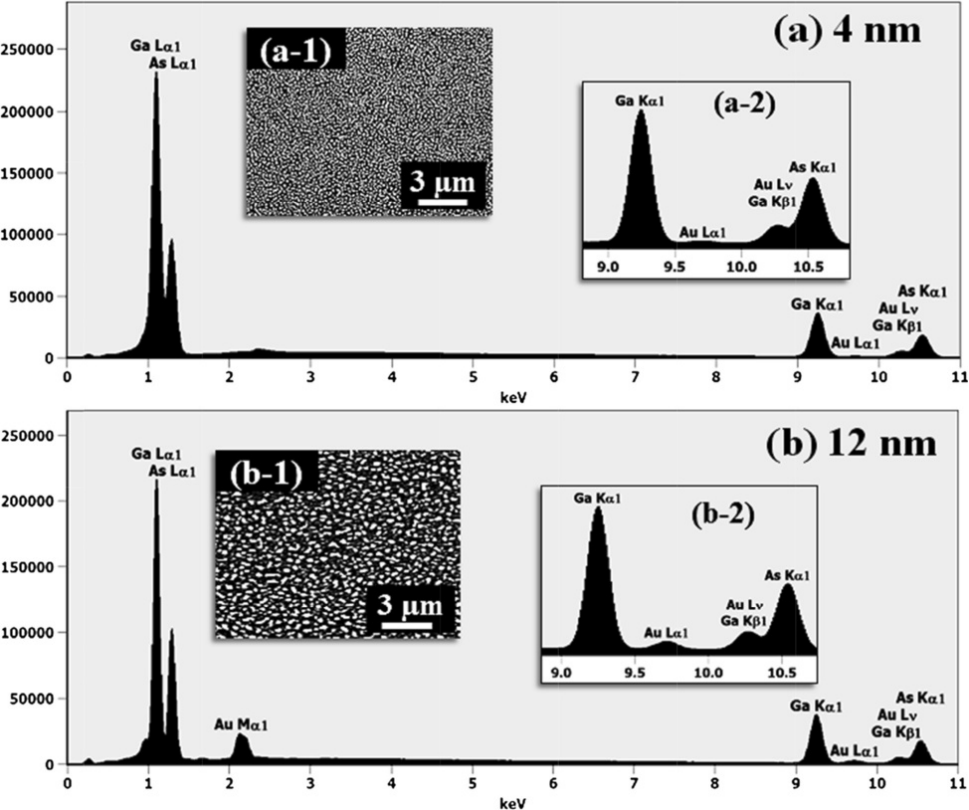
**Surface line profiles and corresponding FFT power spectra. (a-f)** Surface line profiles of the cross sections indicated with the white lines in Figure 7a,b,c,d,e,f of 1 × 1 μm^2^ AFM top views. **(a-1)** - **(f-1)** The corresponding Fourier filter transform power spectra.

**Figure 6:**
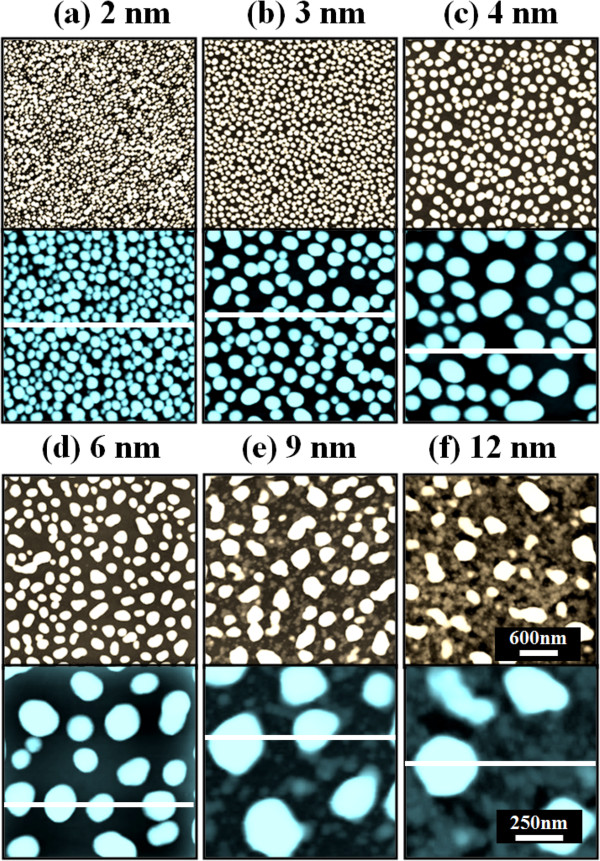
**EDS spectra and SEM images.** Energy-dispersive X-ray spectroscopy (EDS) power spectra of samples with **(a)** 4-nm and **(b)** 12-nm DAs. **(a-1)**, **(b-1)** The corresponding scanning electron microscope (SEM) images. **(a-2)**, **(b-2)** The enlarged spectra between 9 to 11 keV.

Figure 2 shows the self-assembled Au droplets on GaAs (211)B by the systematic variation of the Au DA from 2 to 12 nm with subsequent annealing at 550°C. Figure labels indicate the related DAs. AFM top views (3 × 3 μm^2^) of the corresponding samples are shown in Figure 2a,b,c,d,e,f along with enlarged 1 × 1 μm^2^ images below. The line profiles in Figure 3a-1,b-1,c-1,d-1,e-1,f-1 show the cross sections indicated by the white lines in the 1 × 1 μm^2^ images of Figure 2. The Fourier filter transform (FFT) power spectra shown in Figure 3a-1,b-1,c-1,d-1,e-1,f-1 are transformed from each AFM image. Figure 4a,b summarizes the average height (AH) and the lateral diameter (LD) of the self-assembled Au droplets, and Figure 4c,d shows the average density (AD) of the corresponding samples as well as the RMS surface roughness (*R*_q_) as a function of the DA. The self-assembled Au droplets were fabricated based on the Volmer-Weber growth mode, thus resulting in the initial appearance of round dome-shaped droplets at 2 nm as in Figure 2a [32-34,38]. Once sufficient thermal energy for the surface diffusion is supplied, Au adatoms can be driven to diffuse. As a result of the binding energy between Au adatoms (*E*_a_) being greater than the binding energy between Au adatoms and surface atoms (*E*_i_), the Au droplets can be nucleated from the thin Au film during surface diffusion [39,40]. After the nucleation, nuclei can grow by absorbing nearby adatoms inward as well as merging with other smaller nuclei and thus can form into gradually larger round dome-shaped droplets. After systematic annealing with 2-nm deposition as shown in Figure 2a, dense Au droplets of round dome shapes were synthesized with an AH of 22.5 nm and LD of 86.5 nm, and the AD was 3.2 × 10^10^ cm^-2^ as plotted in Figure 4. When the DA was increased to 3 nm as shown in Figure 2b, the size of droplets was increased by × 1.38 to 31.1 nm for the AH and by × 1.23 to 106.5 nm for the LD as plotted in Figure 4a,b. Meanwhile, the corresponding AD was shapely decreased by × 3.08 from 3.2 × 10^10^ cm^-2^ to 1.04 × 10^10^ cm^-2^ as plotted in Figure 4c. Then at the 4-nm DA, the size of Au droplets was increased by × 1.44 to 44.9 nm for the AH and × 1.33 to 142.4 nm for the LD, and the AD was 3.9 × 10^9^ cm^-2^ which was decreased by × 2.66. Then the trend, namely increased size along with the decreased density, was continuously maintained with the increased DA for 6 to 12 nm, and notably, at 6-nm DA as seen in Figure 2d, droplets began to show slightly irregular shapes without any preferential direction as evidenced by the round FFT power spectrum in Figure 3d-1. The LD measurements were performed along the shorter diameter. When the DA increased from 6 to 12 nm, the AH was further increased from 52.5 to 71.1 nm, the LD was increased from 186.2 to 276.8 nm, and the corresponding AD was dropped to 4.2 × 10^8^ cm^-2^. Overall, with the DA variation from 2 to 12 nm, the AH of the self-assembled Au droplets was increased by × 3.16 from 22.5 to 71.1 nm and the LD was increased by × 3.20 from 86.5 to 276.8 nm as shown in Figure 4a,b. Meanwhile, the corresponding AD was decreased by nearly 2 orders from 3.2 × 10^10^ to 4.2 × 10^8^ cm^-2^. The size of droplets can be increased with decreased density when more amount of material is provided. This evolution of size and density can be a conventional behavior, and it also can be observed with other self-assembled nanostructures [41-44]. The diffusion length (*l*_D_) can be defined as $l_{D}\;=\;\sqrt{\text{Dτ}}$ (where *D* is the surface diffusion coefficient and *τ* is the residence time), and the *D* has a strong proportional dependency on the substrate temperature (*D* ∝ *T*_sub_). Then, driven by a high *T*_sub_, the *l*_D_ can be significantly increased. In a thermodynamic equilibrium system, nanostructures tend to increase their dimensions by absorbing nearby adatoms to lower the surface energy until reaching the equilibrium in order to keep the energy of the whole system in the lowest state. Therefore, when more adatoms exist within the *l*_D_, the increased dimensions of droplets can be expected. In terms of the uniformity, the color pattern of the FFT power spectrum represents the frequency of the height with a directionality. The FFT spectrum with the 2-nm DA in Figure 3a-1 showed a round shape due to the round shape of the droplets. With the 3-nm DA, a smaller core of the FFT pattern was observed due to the reduced height frequency associated with the reduced density in Figure 3b-1 as well as the AFM image in Figure 2b. Then, the FFT patterns in Figure 3c-1,d-1,e-1,f-1 with the increased DAs became smaller and smaller as the frequency of the height became narrower and uniform. In addition, flat tops of droplets were observed with the line profiles of the DAs of 9 and 12 nm in Figures 3e,f and 5e,f. This is in strong contrast with the round dome-shaped droplets at lower DAs. In the case of Si with the increased Au deposition amount, lateral growth of Au nanostructures occurred even with as low as approximately 5-nm DA and finally resulted in the formation of a merged Au layer at approximately 20-nm DA [45]. However, in this experiment, the droplets were still maintained even above 12-nm DA (not shown here). Although it is not very logical to compare GaAs and Si directly due to the different growth conditions such as temperature, from this result, it can be expected that the binding energy between Au adatoms and surface atoms (*E*_i_) is weaker on GaAs surfaces than on Si (111). In other words, with increased DAs, droplets with lateral dimension expansion (coalescence) would require much higher DAs. In terms of the surface roughness (*R*_q_) during the DA variation from 2 to 3 nm, the *R*_q_ was increased from 6.22 to 11.63 nm along with the expansion of the droplet dimensions as shown in Figure 4d. With the gradually increased DAs, the *R*_q_ in Figure 4d showed an increasing trend accompanied with increased droplet dimensions, 6.22 nm for the 2-nm DA and 11.63 for the 3-nm DA, and gradually increased to 24.37 nm at the 9-nm DA. Then, the *R*_q_ was saturated and showed a decreasing trend from there, likely due to the dominance of density decrease over the dimensional increase. Figure 6 shows the EDS spectra of the surface elemental characterization and the related SEM images of 4- and 12-nm samples. Generally, the resulting EDS spectra showed similar spectra for Ga and As with 4- and 12-nm DA as expected. The Kα1 peaks of Ga and As at 9.243 and 10.532 keV and Lα1 peaks of Ga and As at 1.096 and 1.282 keV were observed in Figure 6a,b. However, likely caused by the variation of the DA and the interaction volume of Au with the X-ray, the Au peaks show obvious difference in peak counts as seen in Figure 6a,b. For example, the Mα1 peak at 2.123 keV of the 12-nm sample showed a peak count value of approximately 22,000 while only approximately 5,000 for 4 nm. Also, the Lα1 peak at 9.711 keV showed a clear difference between 4 and 12 nm as shown in Figure 6a-2,b-2.

**Figure 7:**
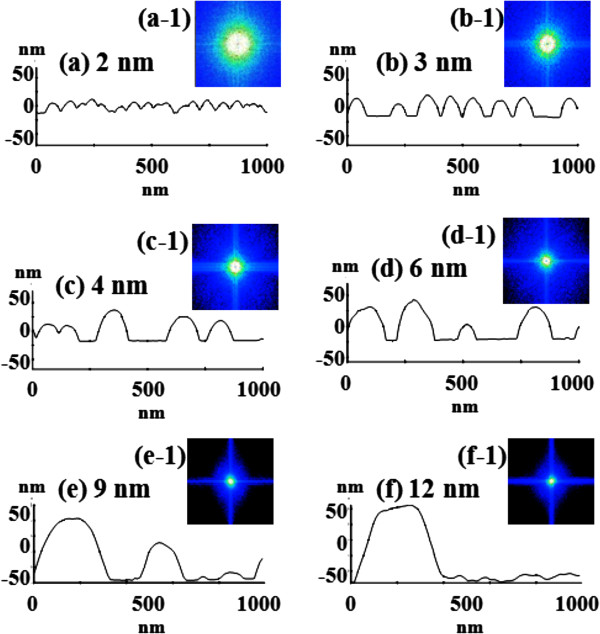
**Evolution of self-assembled Au droplets.** This was induced by the systematic variation of the Au deposition amount from 2 to 12 nm on GaAs (511)B. **(a)** 2 nm, **(b)** 3 nm, **(c)** 4 nm, **(d)** 6 nm, **(e)** 9 nm, and **(f)** 12 nm. Au droplets are presented with AFM top views of 3 × 3 μm^2^ and 1 × 1 μm^2^.

**Figure 8:**
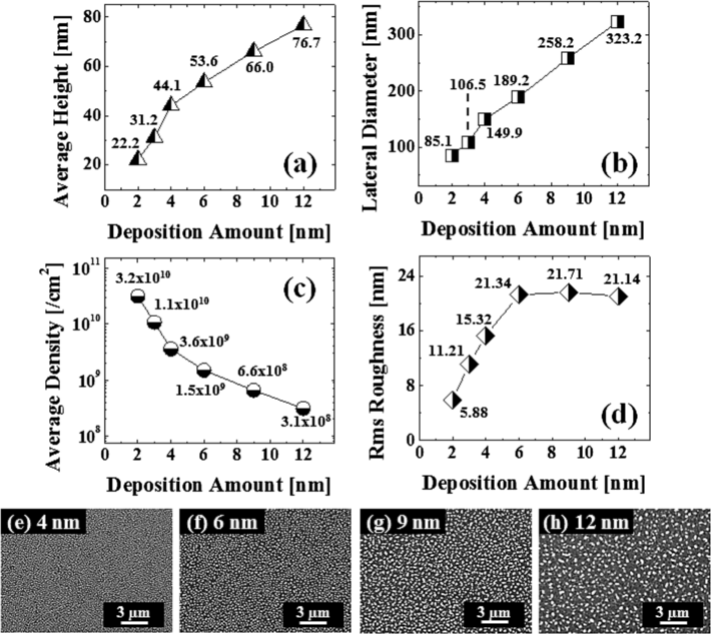
**Summary plots and SEM images.** Summary plots of **(a)** AH, **(b)** LD, **(c)** AD, and **(d)***R*_q_ of the self-assembled Au droplets on GaAs (511)B as a function of DA. **(e**-**h)** SEM images of the resulting Au droplets with the DAs as labeled.

Figure 7 shows the self-assembled Au droplets fabricated on GaAs (511)B, and the results are summarized with the AFM images in Figure 7a,b,c,d,e,f, the line profiles in Figure 5a,b,c,d,e,f, the FFT power spectra in Figure 5a-1,b-1,c-1,d-1,e-1,f-1, the summary plots of the size and density as well as the *R*_q_ in Figure 8a,b,c,d, and finally the SEM images in Figure 8e,f,g,h. Overall, the self-assembled Au droplets on GaAs (511)B showed a similar evolution tendency to that of the GaAs (211)B in terms of the AH, LD, AD, and *R*_q_ as plotted in Figure 8. Namely, the dimensions of the Au droplets including the AH and LD were gradually increased, while the AD was continuously decreased as a function of the DA. For example, while the DA was varied from 2 to 12 nm, the AH of droplets was increased by × 3.45 from 22.2 to 76.7 nm and the LD by × 3.79 from 85.1 to 323.2 nm as clearly shown in Figure 8a,b. The corresponding AD was dropped by × 103.2 from 3.2 × 10^10^ to 3.1 × 10^8^ cm^-2^ as shown in Figure 8c. The FFT pattern started with a relatively larger round shape with 2-nm DA in Figure 5a-1 and gradually became smaller in size with the increased DA for the same reason discussed above as shown in Figure 5b-1,c-1,d-1,e-1,f-1. The *R*_q_ began with 5.88 nm for 2-nm DA and reached 21.71 nm for 9-nm DA, and then the *R*_q_ was decreased to 21.14 nm with 12-nm DA likely due to the dominance of the density decrease.

**Figure 9:**
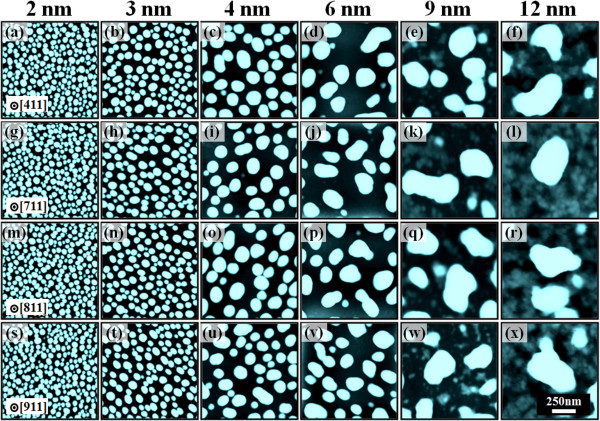
**Au droplet evolution as a function of the DA. (a-x)** Self-assembled Au droplets fabricated by the variation of the Au deposition amount on GaAs (411)B, (711)B, (811)B, and (911)B. The resulting droplets are presented with AFM top views of 1 × 1 μm^2^.

Figure 9 shows the Au droplet evolution as a function of the DA along with the systematic annealing at 550°C on GaAs (411)B, (711)B, (811)B, and (911)B, respectively. As summarized in Table 2, the results in terms of the size and density evolution are quite analogous to the previous two surfaces. For instance, the size of Au droplets on GaAs (411)B was gradually increased (by × 3.16 for AH and × 3.20 for LD), while the AD was progressively decreased by nearly 2 orders during the variation of the DAs from 2 to 12 nm as clearly shown in Table 2. Similar trends of Au droplet evolution on the other three surfaces can be clearly seen in Figure 9 with the comparable magnitude of changes. In general, various GaAs (*n*11)B show distinction in terms of the atom density, dangling bonds, and step density [29-31], and as a result, the resulting self-assembled nanostructures can show different behaviors in terms of size and density and even configurations. However, in this experiment, the difference in the result appeared to be minor. Perhaps, it is because the diffusion length of adatoms has a much stronger dependency on the activation energy and substrate temperature. As mentioned, the diffusion length increases by the square root of the product of the diffusion coefficient and residual time of adatoms ($l_{D}\;=\;\sqrt{\text{Dτ}}$), and the diffusion coefficient is strongly proportional to the substrate temperature (*D* ∝ *T*_sub_). In this experiment, the substrate temperature was fixed at 550°C, and thus the size of the Au droplets can be increased by absorbing Au adatoms within the diffusion length as discussed. Likewise, the diffusion length can also be affected by the variation of atom density, dangling bonds, and step density. However, the difference or the effect induced by the variation of the index to the surface diffusion seems to be relatively smaller as compared to that induced by the substrate temperature [35].

**Table 2:** Summary of the self-assembled droplets on GaAs (211)B, (411)B, (511)B, (711)B, (811)B, and (911)B

	**S**	**DA (nm)**					
		**2**	**3**	**4**	**6**	**9**	**12**
Average height [nm]	(211)B	22.5	31.1	44.9	52.5	67.7	71.1
	(411)B	22.7	30.1	44.9	54.5	69.3	76.8
	(511)B	22.2	31.2	44.1	53.6	66.0	76.7
	(711)B	22.6	33	47.4	56	70.8	77.3
	(811)B	22.8	30.5	44.5	52.7	65.5	74.6
	(911)B	22.3	30.5	44.5	52.7	65.5	74.6
Lateral diameter [nm]	(211)B	86.5	106.5	142.4	186.2	248.8	276.8
	(411)B	89.8	108.1	168.6	214.2	253.2	298.7
	(511)B	85.1	106.5	149.9	189.2	258.2	323.2
	(711)B	87.1	108.9	150.4	222	299	314.5
	(811)B	82.2	105.3	173.7	187.2	292.8	320
	(911)B	81.3	106.4	155.8	213.2	267	304.2
Density [×10^8^ cm^-2^]	(211)B	320	100	39	16	6.1	4.2
	(411)B	320	108	36	15	6.9	3.3
	(511)B	320	110	36	15	6.6	3.1
	(711)B	320	96	28	13	3.9	2.8
	(811)B	304	108	39	16	4.9	2.9
	(911)B	320	112	33	15	5.3	2.8
*R*_q_ [nm]	(211)B	6.22	11.63	15.79	20.76	24.37	19.95
	(411)B	6.64	10.63	16.51	21.48	25.54	21.94
	(511)B	5.88	11.21	15.32	21.34	21.71	21.14
	(711)B	6.97	11.90	15.50	21.07	21.51	18.31
	(811)B	6.68	10.80	17.10	21.32	22.13	20.09
	(911)B	6.80	10.74	16.44	20.50	24.62	18.30

AH, average height; LD, lateral diameter; AD, average density; RMS, root-mean-square roughness (*R*_q_); S, surface indices; DA, deposition amount.

## Conclusions

In this study, the evolution of the self-assembled Au droplets was successfully demonstrated on various GaAs (*n*11)B, where *n* is 2, 4, 5, 7, 8, and 9. With the systematic variation of the DAs from 2 to 12 nm at a fixed annealing temperature of 550°C, the Au droplet growth progressed based on the Volmer-Weber growth mode and the results were methodically investigated with the AFM and SEM images, line profiles, and Fourier filter transform power spectra. In general, along with the gradually increased DAs, the self-assembled Au droplets showed the increased size of the AH and LD, while the AD showed a gradual decreasing tendency. More specifically, both the AH and LD were increased approximately three times while the density was varied around 2 orders of magnitude during the variation of the DAs from 2 to 12 nm. The size and density behavior of the self-assembled Au droplets was discussed based on the theories of kinetics and thermal dynamics. Au droplets exhibited minor index dependency, and this can be likely due to the strong dependency of adatom diffusion on the substrate temperate.

## Competing interests

The authors declare that they have no competing interests.

## Authors' contributions

MS, ML, and JL participated in the experiment design and carried out the experiments. MS, ML, EK, and JL participated in the analysis of data. MS, ML, and JL designed the experiments and testing methods. MS and JL carried out the writing of the manuscript. All authors helped in the drafting and read and approved the final manuscript.
